# Feasibility and efficacy of a novel size adjustable cryoballoon for ablation of atrial fibrillation

**DOI:** 10.1007/s10840-023-01645-2

**Published:** 2023-09-16

**Authors:** Gerrit Frommeyer, Christian Ellermann, Julian Wolfes, Philipp S. Lange, Fatih Güner, Lars Eckardt

**Affiliations:** Department of Cardiology II-Electrophysiology, University Hospital Münster, Albert-Schweitzer-Campus 1, Gebäude A1, 48149 Münster, Germany

**Keywords:** Pulmonary vein isolation, Cryoballoon, Atrial fibrillation

## Abstract

The aim of the present case series was to characterize the feasibility of a novel size adjustable cryoballoon system (PolarX Fit, Boston Scientific, Marlborough, MA, USA). This cryoballoon catheter can be inflated to two different diameters (28 mm and 31 mm) within the same procedure allowing vein adapted PVI. In summary, the novel size adjustable cryoballoon shows similar characteristics as the established versions. The intraprocedural flexibility of balloon size led to employment of the larger variant in the majority of freeze applications. Of note, in all but one procedure, both sizes were employed to ensure optimal occlusion for all veins. This initial series suggests that the size adjustable balloon offers more flexibility of obtain optimal occlusions in particular, in challenging anatomies, including common pulmonary vein ostia.

## Research Letter

Pulmonary vein isolation (PVI) is established and recommended for interventional therapy of atrial fibrillation (AF) [1]. Cryoablation of the pulmonary veins is an established method and has been proven to be non-inferior to radiofrequency ablation in a large randomized trial [2]. This method is characterized by a more extensive use of fluoroscopic guidance and injection of contrast dye in order to ensure an optimal position of the cryoballoon in the antrum of the pulmonary veins. Of note, cryoablation is characterized by a steep learning curve and is in this context comparable to other “single shot” ablation devices [3].

To date, two different cryoablation systems are available. The Arctic Front Advance system (Medtronic, Minneapolis, MN, USA) has been overhauled over the last 15 years and is currently available in two different sizes (23 mm and 28 mm) that cannot be adjusted within the procedure. The latter introduced PolarX system (Boston Scientific, Marlborough, MA, USA) is only available with a diameter of 28 mm. Recent head-to-head comparisons did not reveal significant differences in single- and multi-center analyses [4–6]. The aim of the present case series was to characterize the feasibility of a novel size adjustable cryoballoon system (PolarX Fit, Boston Scientific, Marlborough, MA, USA). This cryoballoon catheter can be inflated to two different diameters (28 mm and 31 mm) within the same procedure allowing vein adapted PVI. There is no change in physical dimensions of catheter or delivery system as the inflation to an increased size is mediated by an electronic update.

The novel cryoballoon was evaluated in 12 consecutive patients with paroxysmal (*n* = 10) or persistent (*n* = 2) atrial fibrillation with a mean age of 68.7 ± 8.4 years. Oral anticoagulation was not interrupted for the procedure. Mean procedure duration (74.3 ± 14.1 min) and fluoroscopy duration (11.99 ± 4.07 min) were comparable to recently published trials implementing the cryoballoon for AF ablation. Pulmonary vein angiography was routinely performed after transseptal puncture. The choice of balloon size was made during the procedure when attempting to occlude each vein with the balloon. Complete isolation of all pulmonary veins could be achieved in 12 of 12 patients. Two of 12 patients presented a common left pulmonary vein ostium. A mean number of 5.5 ± 0.9 applications were delivered per patient. The duration of each freeze was 180 s. Time to isolation (TTI) was monitored, if possible. If TTI was higher than 60 s, a second freeze of 180 s was administered.

**Fig. 1:**
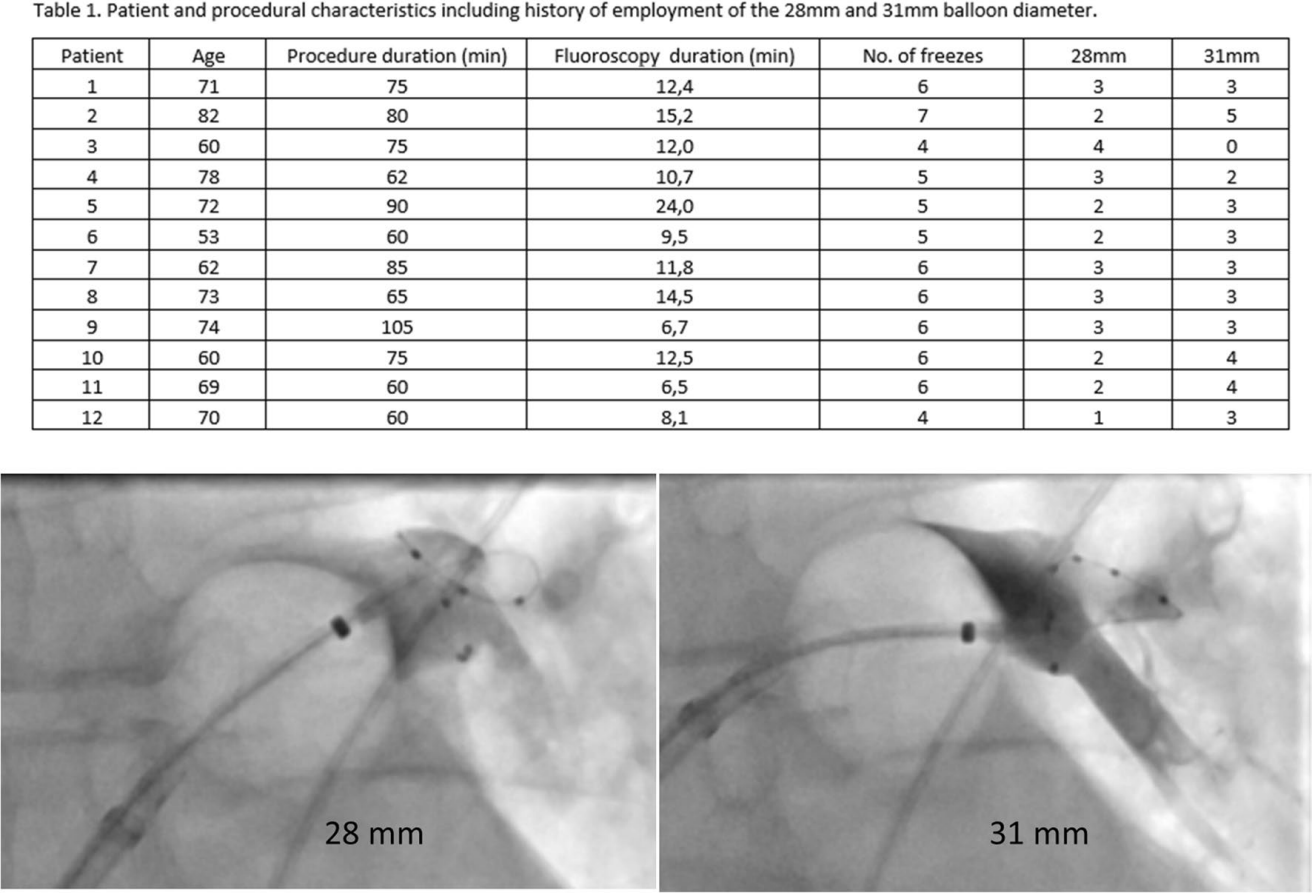
Representative example of cryoballoon configuration and pulmonary vein occlusion with the 28-mm (top) and 31-mm balloon diameter (bottom)

In 55% of applications, the 31-mm diameter was employed, while in the remaining 45% of applications, the 28-mm diameter was sufficient. In one patient, a post-procedural aneurysma spurium was detected and could immediately be effectively compressed. The patient did not require surgical therapy. No pericardial tamponade, stroke, or phrenic nerve palsy were observed in this case series (Fig. 1).

In summary, the novel size adjustable cryoballoon shows similar characteristics as the established versions. The intraprocedural flexibility of balloon size led to employment of the larger variant in the majority of freeze applications. Of note, in all but one procedure, both sizes were employed to ensure optimal occlusion for all veins. This initial series suggests that the size adjustable balloon offers more flexibility of obtain optimal occlusions, in particular, in challenging anatomies including common pulmonary vein ostia. Furthermore, the configuration of the 31-mm balloon may contribute to more antral lesions and may thereby reduce the risk of phrenic nerve palsy. A creation of larger ablation lesions by the larger balloon diameter potentially implies the risk of inducing arrhythmic isthmi by leaving narrow corridors in the posterior wall or peri-mitral region. However, given the only slightly increased diameter of the balloon, this risk seems to be low.
